# Performance of TB immunodiagnostic tests in Eurasian badgers (*Meles meles*) of different ages and the influence of duration of infection on serological sensitivity

**DOI:** 10.1186/1746-6148-5-42

**Published:** 2009-11-17

**Authors:** Mark A Chambers, Sue Waterhouse, Konstantin Lyashchenko, Richard Delahay, Robin Sayers, R Glyn Hewinson

**Affiliations:** 1 TB Research Group, Department of Statutory and Exotic Bacterial Diseases, Veterinary Laboratories Agency Weybridge, New Haw, Surrey KT15 3NB, UK; 2 Veterinary Laboratories Agency Langford, Bristol BS40 5DX, UK; 3 Chembio Diagnostic Systems, Inc., Medford, NY 11763, USA; 4 Central Science Laboratory, Sand Hutton, York YO4 1LZ, UK; 5 Centre for Epidemiology and Risk Analysis, Veterinary Laboratories Agency Weybridge, New Haw, Surrey KT15 3NB, UK

## Abstract

**Background:**

In parts of Great Britain and Ireland, Eurasian badgers (*Meles meles*) constitute a reservoir of *Mycobacterium bovis *infection and a potential source of infection for cattle. *In vitro *diagnostic tests for live badgers are an important component of strategies to control TB in this species. Immunological tests have been developed for badgers, although little is known about the influence of the age of the animal on test performance. To address this, we evaluated the performance of three immunological tests for badgers with respect to the age of the animal: the Brock Test and BrockTB STAT-PAK^® ^serological tests and the recently developed interferon-gamma enzyme immunoassay (IFNγ EIA). Data published elsewhere suggested that seropositivity was associated with more progressive forms of TB in the badger. To gain further evidence for this, we used longitudinal data from a well-studied population of badgers to test for an association between the sensitivity of the Brock Test and the duration of TB infection.

**Results:**

Sensitivity of the two serological tests was approximately 54% for both cubs and adults. Sensitivity of the IFNγ EIA was lower in cubs (57%) compared with adults (85%) when a common cut-off value was used to define test positivity. Taking data from the cubs alone, the IFNγ EIA cut-off value could be adjusted to increase the sensitivity to 71% with no loss in specificity. As a general observation, specificity of all tests was higher in cubs, although only significantly so in the case of the Brock Test. Using logistic regression analysis to adjust for age, sensitivity of the Brock Test was significantly lower at first culture positive event (58%), but increased to >80% as infection progressed.

**Conclusion:**

These data suggest that serodiagnosis could be a valuable tool for detecting a higher proportion of badgers with the greatest probability of transmitting infection. The age category of the badger appeared to exert little influence on the performance of the serological tests. Although data were only available for the IFNγ EIA in a small number of cubs, reduced sensitivity of the test in these individuals suggests a lower cut-off may be needed when testing younger animals.

## Background

Despite attempts to control bovine tuberculosis (TB), by the end of 2007 the average incidence of disease in cattle in Great Britain (GB) had increased nearly 400% over the previous thirteen years [1], causing considerable economic loss to the farming community and high costs to Government. In parts of GB and Ireland, Eurasian badger (*Meles meles*) populations constitute a reservoir of *Mycobacterium bovis *infection and a potential source of infection to cattle [2-4]. Accurate diagnosis of *M. bovis *infection in badgers is an important component of the development of strategies to control TB in this species. Culture of *M. bovis *is still considered the 'gold-standard' diagnostic test. However, confirmation of TB in the badger by culture is particularly insensitive using clinical samples obtained from the live animal; in part due to the intermittent nature of detectable bacterial excretion among infected animals [5]. The sensitivity of culture of necropsy tissues was recently estimated at around 55% using a standard necropsy protocol. More infected animals, particularly adults and those with no visible lesions, were detected using a protocol that included many more tissues, cultured individually, and submitted to an extended culture regime [6]. Against this background, alternative, sensitive *in vitro *diagnostics that can be used to test live animals are required.

The first immunological test for TB infection in the live badger was called the Brock Test; a serum antibody ELISA test directed to a single antigen of *M. bovis*, MPB83, developed over 10 years ago [7]. The Brock Test has traditionally lacked sensitivity (range 40-53%, depending on report) [7-10]. Subsequent developments in badger immunology have resulted in a lateral flow serum antibody test (BrockTB STAT-PAK^®^) [8] and most recently, an Enzyme Immunoassay (EIA) for interferon-gamma (IFNγ) [10], analogous to the BOVIGAM^® ^TB test for cattle. The BrockTB STAT-PAK^® ^is no more sensitive than the Brock Test [8] but is cheaper, quicker, and easier to perform. In contrast, the IFNγ EIA is a more involved test but has the best performance of any test so far developed for the diagnosis of TB in live badgers, with a sensitivity of 80.9% and a specificity of 93.6% [10].

However, few reports stratify the performance of immunodiagnostic tests for TB in animals relative to age. Previous studies using the Brock Test in badgers suggested that it may give a higher rate of false positive reactions (i.e. lower specificity) in cubs/juveniles compared with adults [7,11]. In a similar vein, the BOVIGAM^® ^IFNγ TB test for cattle cannot be used in young animals due to a large proportion of non-specific reactors; a fact attributed to the production of IFNγ by NK cells *ex vivo *in response to mycobacterial antigens, even when calves are uninfected [12]. Therefore we sought to evaluate the sensitivity and specificity of the Brock Test, BrockTB STAT-PAK^®^, and IFNγ EIA in badgers of different age groups.

We reported recently that the sensitivity of the BrockTB STAT-PAK^® ^was significantly higher in animals with more severe TB, characterised by high excretion frequency of *M. bovis *or the presence of visible lesions at necropsy [13]. We inferred that such animals represent more advanced disease and the greatest risk for onward transmission of infection, and proposed that serological tests would be more suitable for detecting such animals. Here we provide supporting evidence for this, as the sensitivity of the Brock Test increased significantly, the longer the badger had been detected as tuberculous.

## Results

### Influence of age of animal on test performance

Table 1 shows the sensitivity and specificity of the badger immunological assays by age category. The sensitivity of the two serological tests was found to be no different between cubs and adults within either test, and was within the ranges reported previously [7-10]. In contrast, the sensitivity of the IFNγ EIA was lower in cubs compared with adults. This difference was not statistically significant, which is likely to relate to the wide confidence intervals around the estimate of the sensitivity of the test in cubs due to the low number of infected cubs available for testing (n = 7). Nonetheless, this result suggests that the cut-off value used to determine positivity in the test might need to be adjusted when applied to cubs.

**Table 1:** Sensitivity and specificity of three immunodiagnostic assays for TB in badger cubs and adults obtained from the RBCT relative to culture of *M. bovis *from necropsy tissues

	Brock Test		BrockTB STAT-PAK^®^		IFNγ EIA	
**Age**	**Sensitivity** **(95% CI, N)**	**Specificity** **(95% CI, N)**	**Sensitivity** **(95% CI, N)**	**Specificity** **(95% CI, N)**	**Sensitivity** **(95% CI, N)**	**Specificity** **(95% CI, N)**

Cub	53.9% (37.1-69.9, 39)	96.6%^a^ (93.5-98.5, 238)	56.4% (39.6-72.2, 39)	96.2% (92.9-98.3, 238)	57.1% (18.4-90.1, 7)	97.5% (86.8-99.9, 40)
Adult	54.7% (49.2-60.1, 340)	93.0%^a^ (91.1-94.7, 817)	49.7% (44.2-55.2, 340)	92.5% (90.5-94.2, 817)	84.6% (69.5-94.1, 39)	92.5% (87.0-96.2, 147)

^a^Significantly different from one another (P = 0.046, Fisher's Exact test of proportions).

**Figure 1:**
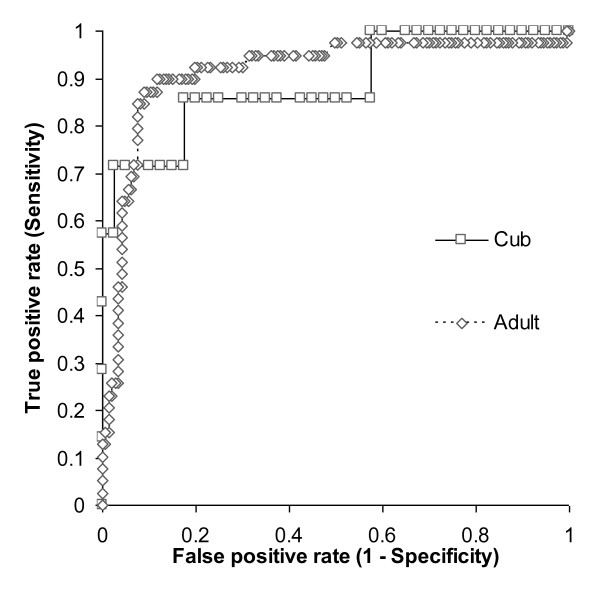
**Receiver Operating Characteristic (ROC) curve analysis for the IFNγ EIA**. The performance of the test in 47 badger cubs and 186 adults is shown, based on TB status determined by culture for *M. bovis*. Each point on the curve represents a different OD value for the test, and is plotted against the sensitivity and specificity corresponding to that OD value as the test cut-off.

To investigate this further we generated non-parametric receiver operating characteristic (ROC) curves from the data (Figure 1). The existing cut-off for the test (0.044) was chosen to maximize the number of correct test results when no differentiation of badger age was made [10]. By ROC analysis, if the test cut-off was reduced to 0.023 when applied to cubs, the sensitivity would increase from 57.1% to 71.4%, whilst still retaining a specificity of 95.0%.

An earlier longitudinal study of the Brock Test using badgers that were captured, sampled, then released as part of on-going ecological and epidemiological studies in Woodchester Park (WP), south-west England [14], indicated that Brock Test positive results in young animals were frequently transient and did not necessarily correspond with *M. bovis *culture positivity [11], leading to the perception that serological tests for TB produce more 'false positive' results in cubs compared with adults. In the WP study, animals were sampled live for *M. bovis *culture and so the true TB status of culture negatives was not known. By using samples from badgers of the Randomised Badger Culling Trial (RBCT) we have been able to readdress this issue, and report that the specificity of the Brock Test was just significantly higher (P = 0.046, Fisher's Exact Test) in cubs compared with adults. The same trend was almost significant for the BrockTB STAT-PAK^® ^(P = 0.053).

### Influence of duration of infection

We previously found that seropositivity is more likely in badgers at more advanced stages of TB [13] and that the Brock Test is more sensitive in detecting badgers with a history of excreting *M. bovis *[5]. To investigate this further with respect to the age of the animal, we evaluated Brock Test results from WP badgers. The BrockTB STAT-PAK^® ^and IFNγ EIA have only recently been introduced in the WP study so insufficient data were available for their inclusion here. Instead two analyses were performed. In the first case, the Brock Test result was analysed with respect to the age category in which the badger was first culture positive. The analysis included animals that were first tested as a cub and were either culture positive then or at a subsequent test. Animals first culture positive as an adult were included only if they were also tested as a yearling. A logistic regression was fitted to available data from 151 animals as a function of age class only. Table 2 shows the distribution of the data and the sensitivity of the Brock Test in badgers of each age category. From the logistic regression analysis, there were no significant differences in Brock Test sensitivity between the age classes (P = 0.266). However, a trend towards increasing sensitivity with the age of the badger at WP was noted.

**Table 2:** Sensitivity of the Brock Test in 151 badgers from WP that were *M. bovis *culture^a ^positive for the first time at the ages shown

	Age category			
**Brock Test**	**Cub**	**Yearling**	**Adult**	**n**

Negative	15	15	36	66
Positive	11	19	55	85
Sensitivity (95% CI)	42.3% (25.1-61.5)	55.9% (39.1-71.4)	60.4% (50.1-70.0)	

^a^From faeces, urine, sputum, pus from abscesses, or bite wound swabs.

**Figure 2:**
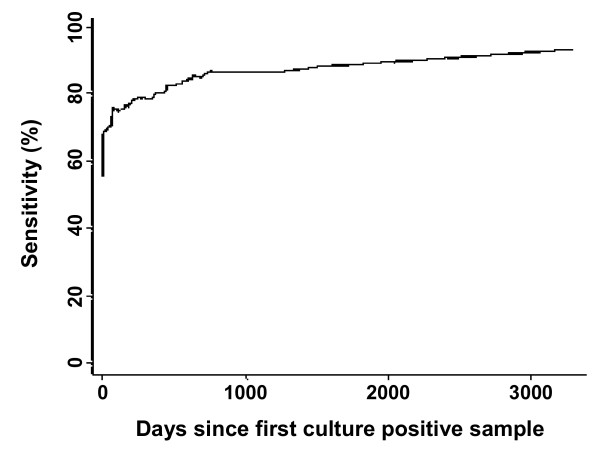
**Brock Test sensitivity increases with duration after first positive culture sample**. Running mean smoothed plot showing how sensitivity of the Brock Test increases with duration (days) after the first positive culture sample.

To test the association between the sensitivity of the Brock Test and the duration of infection, all Brock Test results that corresponded to a sampling event yielding a positive culture were used (n = 279). To facilitate analysis, three 'duration categories' were assigned: (a) the Brock Test result at the time of the first positive culture result; (b) the Brock Test result at the time of a culture positive result within 200 days of the first culture positive result; and (c) the Brock Test result at the time of a culture positive result more than 200 days after the first culture positive result. A logistic regression model fitted to the Brock test results as a function of the age class and duration factors indicated no significant differences in the sensitivity of the Brock Test between age classes (cub, yearling, adult) when adjusted for the 'duration categories' (P = 0.247). This supported the analysis presented in Table 2. In contrast, when adjusted for the age of the badger, the sensitivity of the Brock Test was significantly (P < 0.001) influenced by the length of time the badger had been detected as tuberculous (i.e. the time since its first culture positive result) (Table 3). This is illustrated by Figure 2 that shows the running mean smoothed plot of the Brock test sensitivity against the time in days since the animal's first culture positive sample.

**Table 3:** Sensitivity of the Brock Test in 279 badgers from WP that were detected excreting *M. bovis *for different periods of time

	Duration category^a^			
**Brock Test**	**0**	**1-200**	**>200**	**n**

Negative	66	11	7	84
Positive	85	51	59	195
Adjusted sensitivity^b ^(95% CI)	57.8% (49.5-65.6)	81.8% (68.2-90.3)	88.4% (76.6-94.7)	

^a^Number of days since first culture positive sampling event.

^b^Adjusted for age of badger at time of Brock Test and relative to culture of *M. bovis *from faeces, urine, sputum, pus from abscesses, or bite wound swabs.

## Discussion

In an effort to increase the power of the statistical analyses to detect differences in the performance of the three tests between age classes, we used samples from badger populations where the likelihood of encountering TB positive individuals was known to be high [6,14]. Nonetheless, a far greater proportion of TB negative badgers were available for analysis and the number of cubs confirmed with TB by culture was in some cases very small. As a result, there was low power to detect differences of the order of 10% (power < 50%) for all analyses, except the comparison of specificity between the age classes for the two serological tests. As a consequence, we were unable to detect any difference between the sensitivities of the serological tests (Brock Test and BrockTB STAT-PAK^®^) in cubs and adults. In fact, the sensitivities were very similar. In comparison, the sensitivity of the IFNγ EIA was notably lower in cubs compared with adults (a difference of 27.5%). Since the test cut-off for the IFNγ EIA is based on the difference in magnitude of IFNγ production following stimulation with bovine and avian tuberculins, it could be that the response to bovine tuberculin is simply lower in cubs than adults, possibly because they are likely to be at a less advanced stage of infection than adults. Alternatively, the level of exposure of cubs to environmental mycobacteria such as *M. avium *may be lower than for adults, and hence a smaller difference in the response to bovine and avian tuberculins is more relevant for a positive result (i.e. a lower cut-off is required). Both mechanisms may be working together. We could find no analogous published report, although newborn mice produced lower IFNγ responses than adults to a variety of different antigenic stimuli [15].

Logistic regression analysis of data from WP badgers showed there were no significant differences in Brock Test sensitivity between the age classes. In other words, the sensitivity of the Brock Test was not influenced by the age of the badger *per se*, supporting the results obtained with the RBCT samples. The trend towards increasing sensitivity with the age of the badger at WP would be consistent with the hypothesis that an older animal may harbor *M. bovis *for longer resulting in more opportunity for the infection to progress and bacterial numbers to increase, even if it had failed to yield a positive culture at an earlier stage of life (possibly due to prolonged incubation or containment of infection). This is supported by the observation that when the logistic regression analysis was adjusted for the age of the badger, the sensitivity of the Brock Test was significantly influenced by the length of time the badger had been tuberculous. In other words, the sensitivity of the Brock Test was significantly lower at the first culture positive event, but increased as infection progressed.

By virtue of the larger sample size available for comparing the specificities of the serological tests in cubs and adults, the statistical power to detect a 5% difference between specificity estimates was 80%. In all cases, the specificity of each test varied little between cubs and adults (5% difference or less). Nonetheless, the specificity of the Brock Test was just significantly higher in cubs compared with adults and was nearly so for the BrockTB STAT-PAK^®^. This finding demonstrates that both tests actually produce fewer, or at least no more, 'false positive' results in cubs compared with adults, and concurs with earlier observations made during the initial evaluation of the Brock Test [16].

## Conclusion

Together with previous studies that showed seropositivity is more likely in badgers at more advanced stages of TB [13] and that the Brock Test is more sensitive in detecting badgers with a history of excreting *M. bovis *[5], this study supports the contention that serodiagnosis could be a valuable tool for detecting a higher proportion of badgers with the greatest probability of transmitting infection. It also provides new evidence that test specificity is not apparently influenced by the age of the animal, contrary to previous concerns that higher rates of false positive reactions are seen in younger animals. Elaboration of the most suitable cut-off for the IFNγ test when used in badger cubs should be attempted as data from a larger number of tuberculous cubs become available, so that high sensitivity can be achieved for all age groups.

These results are relevant to researchers and those tasked with formulating policy on badgers and TB, as well as those who use these tests in badger rehabilitation programmes. The findings are useful in interpreting immunological TB test results in badgers of different ages and in the absence of culture confirmation, as well as for determining the relative risks posed by test-positive badgers for transmission of infection. They also highlight the difficulty of performing this type of study in wildlife populations, where the numbers of samples available from animals in different age and disease classes are frequently limited.

## Methods

### Blood samples

Serum and whole-blood samples were obtained from badgers killed as part of the Defra/Independent Scientific Group Randomised Badger Culling Trial [4]. Serum samples were also obtained from badgers captured, sampled, then released as part of an on-going ecological and epidemiological study in Woodchester Park, south-west England [17]. Badgers from the RBCT were subjected to the standard necropsy examination and culture protocol described in [6], with the exception of the 233 from which a whole-blood sample was additionally taken. These were subjected to the detailed necropsy examination and culture protocol described in [6], designed to maximise the certainty associated with the classification of TB status. Resources precluded the conduct of the detailed protocol on all RBCT badgers. The infection status of WP badgers was determined by bacterial culture of clinical samples of faeces, urine, sputum, pus from abscesses, and bite wound swabs as described previously [18]. As these animals were sampled live for *M. bovis *culture the true TB status of culture negatives was not known and hence only samples from culture positive animals were used in this study.

The age classification of each badger was determined from tooth wear characteristics, as previously described [19,20], taking into account each animal's size and weight. Adults were considered to be older than a year (for RBCT badgers) or older than two years (for WP badgers), while cubs were considered to have been born in the year of examination. The age classification of WP badgers included the additional category of 'yearling' for those animals considered to be between 12-23 months of age, reflecting different practices between data recording in both studies.

In total, 1434 serum samples were obtained from RBCT badgers (277 from cubs and 1157 from adults), which were divided into 39 cubs and 340 adults with TB confirmed by culture. In addition, 233 whole-blood samples were obtained from a subset of these badgers (47 from cubs and 186 from adults), which were divided into 7 cubs and 39 adults with TB confirmed by culture. Serum was obtained from 151 culture-positive WP badgers (26 from cubs, 34 from yearlings, and 91 from adults).

Permission was obtained at the time from the Independent Scientific Group on Cattle TB to use samples from the RBCT. Anaesthesia and blood sampling of badgers was carried out under licences issued according to the Animals (Scientific Procedures) Act 1986, following local ethical review.

### Serological tests

Badger serum samples were subjected to the Brock Test, the current indirect ELISA for detection of antibodies to *M. bovis*, according to the published protocol [21], and data reported as OD units. A positive result was taken as any where the OD value exceeded the cut-off value in use at the time the samples were tested. This value changed over the period of testing whenever a new batch of test reagent (antigen, antibody, conjugate) was introduced, according to internal quality control procedures designed to maintain a consistent sensitivity and specificity for the test. The BrockTB STAT-PAK^® ^was developed by Chembio Diagnostic Systems, Inc. using coloured latex-based lateral-flow technology and a cocktail of selected *M. bovis *antigens including ESAT-6, CFP10, and MPB83 in a cassette format [8]. Serum samples were tested as previously described [22]. Results were read 20 minutes after adding sample buffer. Any visible band in the test area of the cassette, in addition to the control line, was considered an antibody positive result, whereas no band in the test area in addition to the visible control line was considered a negative result.

### IFNγ EIA

Whole heparinised badger blood was mixed in 24 well culture plates in a 1:1 ratio with RPMI 1640 medium (Invitrogen) so that each culture (1.5 ml volume) contained 50 units/ml penicillin (Invitrogen), 50 μg/ml streptomycin (Invitrogen), 25 IU/ml heparin (Roche) and either 30 μg/ml of purified protein derivative from *M. bovis *(PPD-B) or 30 μg/ml of purified protein derivative from *M. avium *(PPD-A). Control wells either contained no antigen (negative control) or Pokeweed Mitogen (PWM, Sigma) at 5 μg/ml (positive control). Well contents were mixed by swirling the culture plates on a smooth flat surface, before incubating at 37°C plus 5% CO_2 _for 16-24 h. Following incubation, as much plasma was removed into 96-well culture plates (NUNC) containing 16 IU/ml heparin per well as possible without disturbing the sediment (typically >0.2 ml), and stored at -80°C.

IFNγ was measured in the plasma samples by ELISA as per a published protocol [10] using capture monoclonal antibody 10H6-C1 at 2.5 μg/ml, biotin-labelled monoclonal antibody 11B9 at 2.5 μg/ml, and streptavidin horseradish peroxidase (Vector Laboratories, Inc. CA) at 1:32000. Capture antibody diluted in 50 mM carbonate buffer pH 9.6, was bound to polysorb 96-well EIA plates (Nunc, Denmark) overnight at 4°C. After 4 washes with PBS (pH 7.2) containing 0.05% Tween 20 (PBSTw20), plates were blocked with 200 μl SYNBLOCK (Serotec, UK) for 30 minutes at room temperature and then washed four times with PBSTw20 before the addition of 100 μl/well of plasma. All samples were assayed in duplicate and results were expressed as optical densities (OD). A positive result was taken as any mean OD value in response to stimulation with PPD-B that exceeded the mean response to PPD-A by 0.044, according to the criteria described [10], regardless of the age classification of the badger. The production of IFNγ in response to PWM stimulation was used as an internal control for assay validity.

### Analyses and statistics

Test sensitivity and specificity using blood samples from the RBCT were assessed by comparison with the current gold standard (*M. bovis *culture from necropsy tissue samples). All WP badgers used for analysis were deemed tuberculous on the basis of isolation of *M. bovis *following culture of clinical samples. Since badgers from WP were sampled live, analysis was only made in terms of test sensitivity. As repeat samples were obtained from individual badgers at WP, it was possible to allocate tuberculous animals to a 'duration category' based on the number of days since *M. bovis *was first isolated by culture from a clinical sample.

Receiver Operator Characteristic (ROC) analysis was performed for IFNγ EIA data against TB status determined by culture for *M. bovis*, using Analyse-it software version 1.73 (Analyse-it Software, Ltd). Test of significance between proportions (Fisher's Exact Test) was performed using GraphPad InStat version 3.06 for Windows (GraphPad Software, San Diego California USA, http://www.graphpad.com).

The logistic regression analyses and the running mean smoothed plot were done with STATA software (StataCorp. 2007. *Stata Statistical Software: Release 10*. College Station, TX: StataCorp LP.). The statistical significance of the predictor variables was assessed using Wald tests. Where appropriate, robust variance estimates were used to adjust for the clustering of samples by animal [23].

A P value of less than 0.05 was considered to demonstrate a significant effect.

## Authors' contributions

SW was responsible for generating the immunological test data. MAC and RD were responsible for providing the samples from the RBCT and WP badgers, respectively. KL provided the BrockTB STAT-PAK^® ^tests. MAC devised the study and wrote the manuscript, with contributions from all authors. RS performed the logistic regression analysis and advised on the interpretation of the results together with RD. All authors read and approved the manuscript.
